# Protective Roles for RGS2 in a Mouse Model of House Dust Mite-Induced Airway Inflammation

**DOI:** 10.1371/journal.pone.0170269

**Published:** 2017-01-20

**Authors:** Tresa George, Matthew Bell, Mainak Chakraborty, David P. Siderovski, Mark A. Giembycz, Robert Newton

**Affiliations:** 1 Airways Inflammation Research Group, Snyder Institute for Chronic Diseases, University of Calgary, Calgary, Alberta, Canada; 2 Immunology Research Group, Snyder Institute for Chronic Diseases, University of Calgary, Calgary, Alberta, Canada; 3 Blanchette Rockefeller Neuroscience Institute, West Virginia University, Morgantown, West Virginia, United States of America; Telethon Institute for Child Health Research, AUSTRALIA

## Abstract

The GTPase-accelerating protein, regulator of G-protein signalling 2 (RGS2) reduces signalling from G-protein-coupled receptors (GPCRs) that signal via Gαq. In humans, RGS2 expression is up-regulated by inhaled corticosteroids (ICSs) and long-acting β_2_-adrenoceptor agonists (LABAs) such that synergy is produced in combination. This may contribute to the superior clinical efficacy of ICS/LABA therapy in asthma relative to ICS alone. In a murine model of house dust mite (HDM)-induced airways inflammation, three weeks of intranasal HDM (25 μg, 3×/week) reduced lung function and induced granulocytic airways inflammation. Compared to wild type animals, *Rgs2*^-/-^ mice showed airways hyperresponsiveness (increased airways resistance and reduced compliance). While HDM increased pulmonary inflammation observed on hematoxylin and eosin-stained sections, there was no difference between wild type and *Rgs2*^-/-^ animals. HDM-induced mucus hypersecretion was also unaffected by RGS2 deficiency. However, inflammatory cell counts in the bronchoalveolar lavage fluid of *Rgs2*^-/-^ animals were significantly increased (57%) compared to wild type animals and this correlated with increased granulocyte (neutrophil and eosinophil) numbers. Likewise, cytokine and chemokine (IL4, IL17, IL5, LIF, IL6, CSF3, CXCLl, CXCL10 and CXCL11) release was increased by HDM exposure. Compared to wild type, *Rgs2*^-/-^ animals showed a trend towards increased expression for many cytokines/chemokines, with CCL3, CCL11, CXCL9 and CXCL10 being significantly enhanced. As RGS2 expression was unaffected by HDM exposure, these data indicate that RGS2 exerts tonic bronchoprotection in HDM-induced airways inflammation. Modest anti-inflammatory and anti-remodelling roles for RGS2 are also suggested. If translatable to humans, therapies that maximize RGS2 expression may prove advantageous.

## Introduction

Asthma is typically an allergic inflammatory disease of the airways characterized by chronic inflammation, pulmonary eosinophilia, airways hyperreactivity (AHR) and mucus hypersecretion [1]. While allergens, such as house dust mite (HDM), are major triggers for allergic asthma [2], inhaled glucocorticoids, or corticosteroids (ICS), reduce airways inflammation and are the most effective drugs in the treatment of mild to moderate asthma [3]. However, in more severe disease, as well as in disease exacerbations, neutrophilic inflammation is common and ICSs show lower clinical efficacy [1, 3]. Treatment guidelines therefore recommend the use of inhaled long-acting β_2_-adrenoceptor agonist/ICS combination therapy, which produce greater clinical benefit compared to increasing the dose of ICS [3].

Glucocorticoids act on the glucocorticoid receptor (gene *NR3C1*) to reduce expression of numerous inflammatory genes, and this explains their profound anti-inflammatory effects of ICS in mild-to-moderate asthma [1, 3]. Mechanistically, how this effect occurs is the subject of ongoing debate, but is likely to involve multiple repressive mechanisms [4–6]. These include transrepression, whereby GR directly interacts with the inflammatory transcription factors responsible for inducing expression of inflammatory genes. In a second form of transrepression, GR may bind negative glucocorticoid response elements (GREs) to repress gene expression [7]. Conversely, transcriptional activation, or transactivation, by GR results in the up-regulation of multiple genes that can repress inflammatory gene expression and/or produce potential benefits in asthma [3, 5]. While transcriptional activation occurs in the human airways post-ICS [8, 9], it is likely that GR transrepression and transactivation operate in concert to repress inflammatory gene expression [10].

Regulator of G-protein signalling 2 (RGS2) is a Gα-directed, GTPase-accelerating protein that reduces heterotrimeric G-protein-dependent signalling and preferentially targets Gαq [11, 12]. Since heterotrimeric G-proteins containing Gαq couple to multiple GPCRs (e.g. the histamine H_1_ receptor, M_1/3_ muscarinic receptors, leukotriene receptors, or protease-activated receptors (PARs)) that play key roles in asthma pathogenesis, a role for RGS2 as an “anti-asthma” gene is predicted. Indeed, while RGS2 expression is reduced in asthmatics, airways smooth muscle (ASM) from mice lacking RGS2 expression shows enhanced contractile responses and *Rgs2*^-/-^ animals have AHR [13, 14]. However, RGS2 expression is induced by glucocorticoids in human pulmonary A549 cells and primary human airways epithelial cells [15–17]. Furthermore, RGS2 expression is strongly induced by stimuli, including β_2_-adrenoceptor agonists, which activate the classical cAMP-dependent protein kinase (PKA) cascade [18–20]. Thus, in human ASM cells and in bronchial airway epithelial BEAS-2B cells, LABAs strongly induce RGS2 expression in a manner that is profoundly enhanced and prolonged by glucocorticoids [13, 17]. Since this enhanced RGS2 expression attenuates increases in intracellular Ca^2+^ ([Ca^2+^]_i_) induced by histamine and methacholine, a beneficial bronchoprotective role for RGS2 in asthma is indicated [13, 17]. Furthermore, synergy between LABAs and glucocorticoids occurs in primary human epithelial cells and RGS2 expression is also induced in the human airways post-ICS inhalation [9, 17]. Since agonists acting at Gαq-coupled receptors can increase the expression of pro-inflammatory cytokines, for example IL6 and IL8, from various cell types, including epithelial and ASM cells [21, 22], a possible anti-inflammatory role for RGS2 expression is hypothesized [17], but currently untested *in vivo*.

In mice, RGS2 expression occurs in ASM and the lungs, but unlike in human cells, mouse RGS2 is not regulated by glucocorticoid [13]. Nevertheless, the ability to show RGS2 expression in the mouse lung combined with the availability of *Rgs2*^-/-^ animals allows functional roles for RGS2 to be tested in mouse models of airways inflammation [23, 24]. Intranasal HDM administration produces allergic airway inflammation and AHR in mice [25–28]. Furthermore, proteases, including those present in HDM, can induce inflammation and reduce lung function via PAR2, which is Gαq-coupled and implicated in murine allergic responses to HDM [2, 29–34]. Therefore, a murine model of HDM-induced airways inflammation was used to test the primary hypotheses that loss of RGS2 expression reduces lung function and worsens inflammation.

## Materials and Methods

### Nomenclature

Unless otherwise indicated, genes, mRNA and protein are referred to using their official NCBI gene symbol. Mouse genes and proteins are referred to according to the Mouse Genome Informatics website (http://www.informatics.jax.org/). Mouse genes are italicised in lower case, with an upper case first letter, whereas the expressed products (protein and, for consistency, mRNA) are provided as non-italicised upper case letters.

### Animals and exposure protocols

Wild type female C57BL/6 mice were purchased from Charles River Laboratories (Wilmington, MA, USA) and housed in the Mouse Single Barrier Unit at the University of Calgary prior to use at ages 10–12 weeks. Alternatively, *Rgs2* wild-type, and knockout (*Rgs2*^−/−^) mice, on a C57BL/6 background, were bred in-house through *Rgs2*^+/−^ × *Rgs2*^+/−^ crosses at the Clara Christie Centre for Mouse Genomics (CCCMG), University of Calgary prior to use at ages 10–12 weeks. These animals were previously re-derived from a stock donated by Dr. Scott Heximer (University of Toronto) [23, 24]. Ear punches were used for genotyping following liberation of DNA by the MicroLYSIS protocol (Gel Company, Inc.; San Francisco, CA). TaqMan PCR (Applied BioSystems; Foster City, CA) was performed using the allele-specific primers and probes in S1 Table.

As described by Gregory *et al*. [27], mice were anesthetized with isoflurane prior to intranasal (*i*.*n*.) administration of either 25 μl phosphate-buffered saline (PBS) (control) or 25 μl (1 mg/ml in PBS) of purified HDM, *Dermatophagoides pteronyssinus* (*Der P1*), extract (Greer Laboratories, Lenoir, NC, USA). In initial experiments, mice were exposed to 25 μg HDM *i*.*n*., 3 and 5 times per week, as well as with 50 μg HDM *i*.*n*. 3 times per week. In each case, the total cell counts in the bronchoalveolar lavage (BAL) fluid were similar and 25 μg HDM 3 times per week was selected for further analyses. Mice were collected 18–24 h after the last HDM exposure for lung function analysis, BAL fluid collection and harvesting of lungs. Prior to the main studies, pilot experiments (n = 5 animals per group) were undertaken to explore the effect of 1, 2, 3 and 5 weeks of inhaled HDM exposure on core parameters of lung inflammation. Analysis of total BAL fluid cell counts, hematoxylin and eosin (H&E) and periodic acid-schiff (PAS) stained lung sections revealed total inflammatory cells in the BAL fluid, lung inflammation and mucus hypersecretion that were maximal following 3 weeks of HDM exposure. Three weeks of inhaled HDM exposure was therefore selected for analyses comparing wild type and *Rgs2*^-/-^ animals.

Protocols were approved by University of Calgary Animal Care Committee according to the Canadian Council for Animal Care guidelines. Mice were age- and sex-matched between the different genotype groups and treatments.

### Lung function analysis

Following induction of anesthesia with sodium pentobarbital (50 mg/kg *i*.*p*.) (CEVA Santé Animale, Libourne, France) and ketamine hydrochloride (90 mg/kg *i*.*m*.) (Wyeth, St Laurin, QC, Canada), animals were tracheostomized and connected to a SciREQ flexivent small animal ventilator (SciReq, Montreal, Ontario). Animals were challenged with aerosolized PBS followed by increasing half-log concentrations (3 mg/ml to 300 mg/ml) of methacholine made up in PBS administered via an in-line nebulizer. Measurement of resistance and compliance was performed using a snapshot 150 perturbation, as described [35].

### Immunohistochemistry

Following inflation and fixing with buffered formalin, the left lung was embedded in wax. Serial, 4 μm, sections were adhered to positively charged slides (Fisher Scientific, Nepean, Ontario, Canada) prior to staining with either with H&E or PAS. Slides were visualised by light microscopy.

H&E stained sections were analyzed using a semi-quantitative scoring system to evaluate the fraction of the airway that was occupied by inflammatory cell infiltrates: 4 = robust inflammation (more than 50% of airway circumference surrounded by inflammatory cell infiltrates); 3 = moderate inflammation (25–50% of airway circumference surrounded by inflammatory cell infiltrates); 2 = mild inflammation (10–25% of airway circumference surrounded by inflammatory cell infiltrates); 1 = minimal inflammation (<10% of airway circumference surrounded by inflammatory cell infiltrates) and a score of 0 = no inflammatory cell infiltrates. Numerical data were averaged from the 3 sections taken from each animal. ASM thickness was measured on H&E stained sections at 4 points around the circumference of the bronchiole by constructing a line perpendicular to the airway circumference and using Image J software to measure the distance from the outer edge to the inner edge of the ASM layer. An average of the 4 measurements was recorded for one bronchiole/section and all measurements were made by an investigator blinded to the study treatments.

PAS-stained sections were scored using a semi-quantitative scoring system where a score of 4 indicated strong staining (more than 75% of the airway epithelium PAS positive), 3 = moderate staining (50–75% of the airway epithelium PAS positive), 2 = mild staining (25–50% of the airway epithelium PAS positive), and a score of 1 = minimal staining (less than 25% of airway epithelium PAS positive). Numerical data were averaged from the 3 sections taken for each animal. Morphometric quantification was also performed on PAS stained sections to quantify the percentage area of the epithelial layer that was positively stained for mucus. Analysis was performed using Image J software to trace a line along the basal border of the epithelium prior to selecting the epithelial cell layer. The percentage of PAS positive area was determined following the application of threshold values for hue, saturation and brightness values. Threshold values were established prior to the analysis to enable identification of all positively stained tissues. These values were then kept constant for analysis of all sections. All the measurements were analysed by an investigator blinded to the tissue codes.

### Cell counts and cytokine release in the BAL fluid

Lungs were flushed out with 3 × 0.5 ml of ice cold PBS. Following centrifugation, BAL fluid was frozen at -80°C for later analysis of cytokines. Cell pellets were resuspended in 1 ml PBS for counting of total cells. Cells were also spun onto glass slides prior to staining with Diff-Quick (Canadawide; Ottawa, ON). Differential cell counts were performed on at least 400 cells/slide and macrophage, eosinophil, neutrophil and lymphocyte percentages converted to absolute numbers using the respective total cell counts.

Cytokine and chemokine levels in the BAL fluid supernatants were quantified using a Mouse Cytokine/Chemokine multiplex Luminex Array (Eve Technologies, Calgary, AB, Canada). Limits of detection were 0.64 pg/ml for all analytes, except for CCL2, CCL3, CCL5, CXCL2, CSF2, IL1B, IL9 and TNF where the limit of detection was 3.2 pg/ml and CCL4 where the limit was 16 pg/ml. Where values fell below the limit of detection, the limit of detection was adopted for downstream analyses. One HDM-treated wild type mouse showed release of cytokines/chemokines into the BAL fluid that was up to 4 standard deviations from the mean and was excluded from the analysis as a statistical outlier.

### RNA extraction and qPCR

Tissue from frozen right lung lobe was disrupted in a TissueLyser LT (Qiagen; Valencia, CA) and RNA prepared by the RNeasy Plus method (Qiagen). Reverse-transcription was performed on 0.5 μg total RNA with qScript kits (Quanta: Gaithersburg, MD). Gene expression was analyzed by qPCR using SYBR GreenER (Life Technologies; Burlington, OH) chemistry using the primers in S1 Table and StepOne Plus or 7900HT PCR instruments (Applied BioSystems). All samples were analyzed in duplicate and relative standard curves for target genes were constructed using a serial cDNA dilution. Results were normalized to the housekeeping mRNA GAPDH.

### Western blot analysis

Tissue from frozen right lung lobe was homogenized into Laemmli buffer prior to size fractionation by SDS-PAGE and immunodetection of RGS2 and GAPDH as previously described [13, 17].

### Statistical analysis

Data from *N* animals are expressed as mean ±SE unless indicated otherwise. Graphing and statistics were performed using GraphPad Prism v6 (Graphpad Inc., La Jolla, CA). Significance was tested by Mann–Whitney U test unless otherwise stated. The null hypothesis was rejected where: * *P* < 0.05, ** *P* < 0.01 or *** *P* < 0.001.

## Results

### Effect of HDM treatment on lung function in wild type and *Rgs2*^-/-^ animals

**Fig 1:**
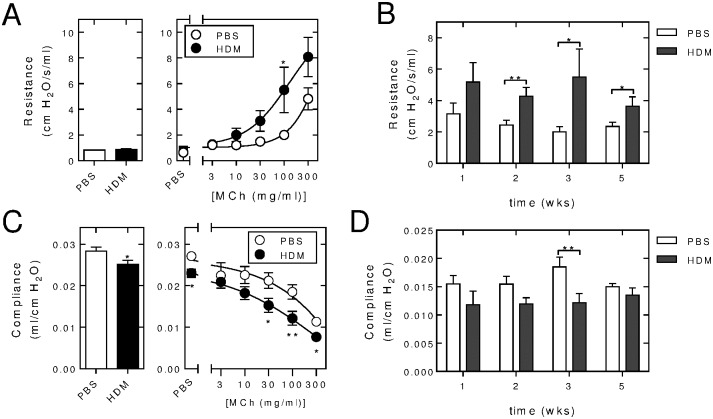
Effect of *i*.*n*. HDM instillation on lung function. A & C, Wild type C57/Bl6 mice were subjected to *i*.*n*. instillation of PBS or HDM (25 μg) 3 times/week for 3 weeks after which lung function was measured using a Flexivent apparatus. Airways resistance (cm H_2_0/s/ml) and compliance (ml/cm H_2_O) are shown at base line (left panels) and following inhaled challenge with PBS or increasing concentrations of methacholine (MCh) as indicated. Data (*N* = 6 for PBS- and 8 for HDM-exposed groups) are plotted as means ± SE. B & D, Wild type C57/Bl6 mice were subjected to *i*.*n*. exposure with PBS or HDM (25 μg) 3 times/week for the indicated number of weeks prior to lung function analysis. Resistance and compliance (*N* = 4–12) following MCh (100 mg/ml) challenge are plotted as means ± SE. Significance was tested between PBS and HDM treatments by unpaired Mann Whitney U test. * *P* < 0.05, ** *P* < 0.01.

PBS or HDM (25 μg) was administrated *i*.*n*., 3 times per week, for 1, 2, 3 and 5 weeks to wild type mice prior to analysis of lung function. Resistance and compliance measurements are shown (Fig 1). At 3 weeks, baseline resistance was similar in PBS- and HDM-exposed animals (Fig 1A, left panel). Aerosolized administration of methacholine produced a dose-dependent increase in lung resistance and in each case this was greater in the HDM-exposed animals when compared to mice given *i*.*n*. PBS for 3 weeks (Fig 1A, right panel). This effect was significant at 100 mg/ml methacholine, a dose that revealed the greatest absolute differential between the two treatment groups. This methacholine concentration was therefore used to compare lung function for each of the 1, 2, 3 and 5 weeks’ exposure periods (Fig 1B). Lung resistance in the HDM-exposed animals, relative to the PBS control, was significantly increased at 2, 3 and 5 weeks. At 3 weeks of HDM exposure, lung compliance at baseline and following PBS challenge was modestly reduced relative to control animals (Fig 1C). With increasing doses of methacholine, there was a further dose-dependent reduction in lung compliance that, with the higher-dose methacholine challenges, was significantly lower in HDM-exposed mice compared to control animals (Fig 1C). Using 100 mg/ml methacholine for comparisons with increasing time, revealed lower compliance in the HDM-exposed mice relative to animals given PBS (Fig 1D). This effect was significant at 3 weeks.

**Fig 2:**
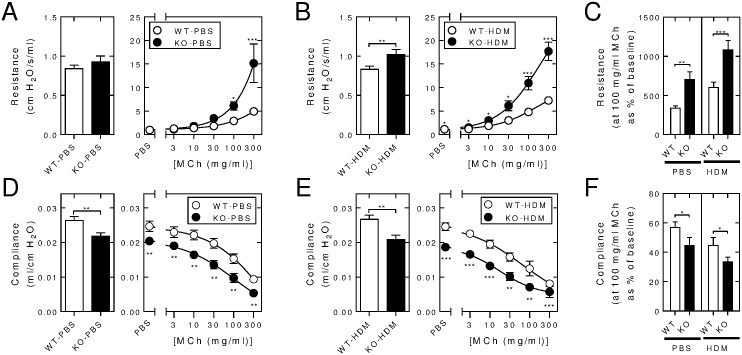
Comparison of lung function between wild type and *Rgs2*^-/-^ mice following *i*.*n*. instillation of either PBS or HDM. Wild type (WT) and *Rgs2*^-/-^ (KO) mice were subjected to *i*.*n*. instillation of PBS (25 μl), 3 times/week for 3 weeks after which lung function was measured using a Flexivent apparatus. A, Airways resistance (cm H_2_0/s/ml) and, D, compliance (ml/cm H_2_O) are shown at base line (left panels) and following inhaled challenge with PBS or increasing concentrations of methacholine (MCh) as indicated. Data (*N* = 16 for wild type and 15 for *Rgs2*^-/-^) are plotted as means ± SE. Wild type and *Rgs2*^-/-^ mice were subjected to *i*.*n* exposure to HDM (25 μg) 3 times/week prior to lung function analysis. B, resistance and, E, compliance at baseline and following inhaled challenge with PBS or increasing concentrations of MCh as indicated are shown. Data (*N* = 27 for wild type and 20 for *Rgs2*^*-/-*^) are plotted as mean ± SE. For each animal, resistance and compliance measurements were expressed as a percentage of baseline. C, Resistance and, F, compliance data from panels A, B, D and E are shown for 100 mg/ml MCh as a percentage of baseline and plotted as means ± SE. Significance was tested between wild type and *Rgs2*^-/-^ groups by unpaired Mann Whitney U test. * *P* < 0.05, ** *P* < 0.01, *** *P* < 0.001.

*Rgs2*^-/-^ and wild-type littermates were subjected to *i*.*n*. PBS or HDM (25 μg) 3 × per week for 3 weeks prior to lung function analysis (Fig 2). Baseline resistance was similar between the PBS-exposed wild type and *Rgs2*^-/-^ animals (Fig 2A, left panel). Methacholine challenge produced dose-dependent increases in resistance that were significantly more exaggerated in the *Rgs2*^-/-^ animals. As described in Fig 1A, 3 weeks of HDM exposure enhanced the methacholine-induced increases in lung resistance in wild type animals (Fig 2A and 2B). HDM-exposed, *Rgs2*^-/-^ animals displayed a modest, but significantly, elevated basal resistance when comparted to wide-type mice (Fig 2B, left panel). This elevation in resistance in the *Rgs2*^-/-^ animals was maintained with the aerosolized PBS challenge and was enhanced in the HDM-exposed *Rgs2*^-/-^ animals at all methacholine doses (Fig 2B, right panel). Furthermore, taking the baseline for each animal as 100% revealed that while HDM exposure produced a significant (*P* < 0.05, ANOVA with a Dunn’s post test) percentage increase in resistance in wild type animals, the PBS- and HDM-exposed *Rgs2*^-/-^ animals also showed a greater percentage increase than the wild type animals for each exposure group (Fig 2C). Thus, the absence of RGS2 expression increased basal airways resistance in HDM-exposed mice and produced both heightened and exaggerated increases in airways resistance in response to methacholine challenge of both PBS- and HDM-exposed animals.

While the data in Fig 1D showed that HDM reduced lung compliance in wild type animals, comparing *Rgs2*^-/-^ animals to wild-type animals, revealed significantly reduced compliance following both the 3 week intranasal PBS and the HDM exposure protocols (Fig 2D & 2E). In each case, there was a loss in baseline compliance in the *Rgs2*^-/-^ animals compared to their wild type counterparts. Likewise, the dose-dependent loss of compliance produced by aerosolized methacholine was significantly greater in the *Rgs2*^-/-^ mice compared to the wild-type animals in both the PBS- and HDM-exposed groups (Fig 2D & 2E). Furthermore, expressing each compliance measurement as a percentage of baseline for each animal reveals that even with the loss of compliance that occurred in the *Rgs2*^-/-^ animals, the percentage loss of compliance following methacholine challenge for each exposure group was also significantly greater with the *Rgs2*^-/-^ mice (Fig 2F). These data show that mouse RGS2 plays a role in reducing absolute lung stiffness and the rate of increase in agonist-induced stiffness in both PBS- and HDM-treated animals.

### Lung inflammation and airways histology in wild type and *Rgs2*^-/-^ animals

**Fig 3:**
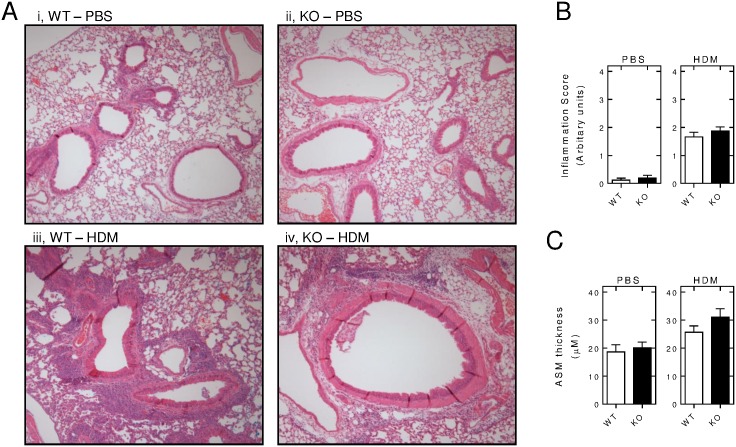
Effect of RGS2 deficiency on HDM-induced lung inflammation. Wild type (WT) and *Rgs2*^-/-^ (KO) mice were subjected to *i*.*n*. instillation of PBS or HDM (25 μg) 3 times/week for 3 weeks. Lungs were dissected, inflated and fixed in 10% formalin and embedded in wax. A, Sections were stained with H&E to allow visualization of inflammatory cell influx. B, Sections were scored using a semi-quantitative scoring system to evaluate the fraction of the airway that was occupied by inflammatory cell infiltrates: 4 = robust inflammation (more than 50% of airway circumference surrounded by inflammatory cell infiltrates); 3 = moderate inflammation (25–50% of airway circumference surrounded by inflammatory cell infiltrates); 2 = mild inflammation (10–25% of airway circumference surrounded by inflammatory cell infiltrates); 1 = minimal inflammation (<10% of airway circumference surrounded by inflammatory cell infiltrates) and a score of 0 = no inflammatory cell infiltrates. Numerical data were averaged from the 3 sections taken/animal. Data (*N* = 17 PBS-exposed wild type and 15 *Rgs2*^-/-^, *N* = 29 HDM-exposed wild type and 24 *Rgs2*^-/-^) are plotted as means ± SE. C, Image J software was used to measure the cross-sectional thickness of the ASM layer at 4 distinct regions surrounding the airway lumen. Data (*N* as in B) are plotted as means ± SE.

Following 3 weeks of PBS exposure, H&E staining of lung sections revealed normal mouse lung morphology both in the wild-type and the knock-out animals (Fig 3A and 3B). In wild-type animals, 3 weeks of HDM-exposure produced lung inflammation, with widespread inflammatory infiltrates around the airways and many of the vessels (Fig 3A and 3B). A similar effect was observed in the 3 week HDM-exposed *Rgs2*^-/-^ animals and this could not be distinguished from the effects observed in the wild-type mice (Fig 3A and 3B).

Examination of the gross morphology suggested that there may be differences in the thickness of the ASM underlying the airways. To examine this possibility, ASM thickness was measured; however, testing of the primary hypothesis did not support a difference in ASM thickness between wild-type and *Rgs2*^-/-^ animals exposed to PBS (Fig 3C, left panel). Following HDM instillation, there was a trend towards increased ASM thickness in the *Rgs2*^-/-^ animals compared to the wild-type controls, but this failed to reach significance (Fig 3C, right panel). Although not a primary end-point, multiple comparison testing (ANOVA with a Dunn’s post test) showed that the increased ASM thickness observed in the HDM-exposed *Rgs2*^-/-^ animals was significantly greater (*P* < 0.01) than in the wide-type animals instilled with PBS. These data therefore suggest a modest protective of RGS2 against remodelling events leading to increased ASM mass in the context of HDM-induced inflammation of the airways.

### Effect of RGS2 deficiency on HDM-induced mucus secretion

**Fig 4:**
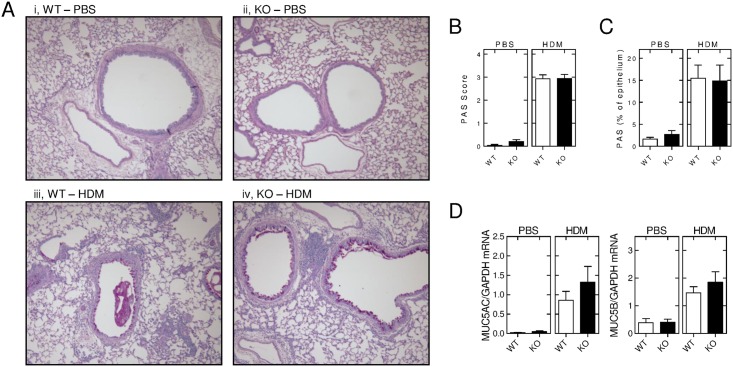
Effect of RGS2 deficiency on HDM-induced mucus secretion. Wild type (WT) and *Rgs2*^-/-^ (KO) mice were subjected to *i*.*n*. instillation of PBS or HDM (25 μg) 3 times/week for 3 weeks. A, Lungs were dissected, inflated and fixed in 10% formalin and embedded in wax. Sections were stained with PAS to allow visualization of mucus. B, PAS-stained sections were scored using a semi-quantitative scoring system where a score of 4 indicated strong staining (more than 75% of the airway epithelium PAS positive), 3 = moderate staining (50–75% of the airway epithelium PAS positive), 2 = mild staining (25–50% of the airway epithelium PAS positive), and a score of 1 = minimal staining (less than 25% of airway epithelium PAS positive). Data (*N* = 17 for PBS-exposed wild type and 15 for *Rgs2*^-/-^, N = 29 for HDM-exposed wild type and 24 for *Rgs2*^-/-^) are plotted as means ± SE. C, Image J software was used to measure the amount of mucus secreted from the airway epithelium and all measurements were made by an investigator blinded to the study treatments. Data (all *N* = 8) are plotted as means ± SE. D, RNA was extracted from the right lungs and mucin gene expression analyzed by qPCR. Data (*N* = 23 for PBS-exposed wild type and 22 for *Rgs2*^-/-^, N = 34 for HDM-exposed wild type and 29 for *Rgs2*^-/-^), normalized to GAPDH, are plotted as means ± SE.

Analysis of PAS-stained lung sections showed little or no evidence of mucus production in either wild type or *Rgs2*^-/-^ animals following 3 weeks of PBS inhalation (Fig 4A, panels i and ii). After 3 weeks of HDM inhalation, there was a dramatic increase in the number of PAS positive, i.e. mucus secreting, cells within the epithelial cell layer (Fig 4A, panels iii and iv). This change was reflected by a substantial increase in the semi-quantitative PAS score (Fig 4B). Close inspection of the epithelial cell layer revealed that in many sections, virtually all the cells that reached the apical (lumen) surface were involved in mucus production and overall ~15% of the epithelial cell layer, by area, was PAS positive (Fig 4C). However, this increase in PAS staining was not modified in the *Rgs2*^-/-^ animals compared to wild-type animals. This is reflected in both the overall PAS score and the morphometric analysis of % PAS staining in the epithelial cell layer. To further interrogate possible effects of RGS2 loss on this mucus hyper-secretion induced by HDM, qPCR was performed for MUC5AC and MUC5B mRNA. As with the PAS staining, only low levels of expression were apparent in the PBS-exposed animals and this was not different between the wild type and *Rgs2*^-/-^ animals (Fig 4D). Following 3 weeks of HDM exposure, expression of MUC5AC and MUC5B mRNAs were elevated by ~37 and ~4 fold in the wild-type and *Rgs2*^-/-^ animals, respectively (Fig 4D). While there was a slight trend towards further increased expression in the *Rgs2*^-/-^ animals, this was not significant. Taken together, the data confirm a profound HDM-induced mucus hyper-secretion that was not modified by RGS2 deficiency.

### BAL fluid inflammatory cell recruitment in wild type and *Rgs2*^-/-^ animals

**Fig 5:**
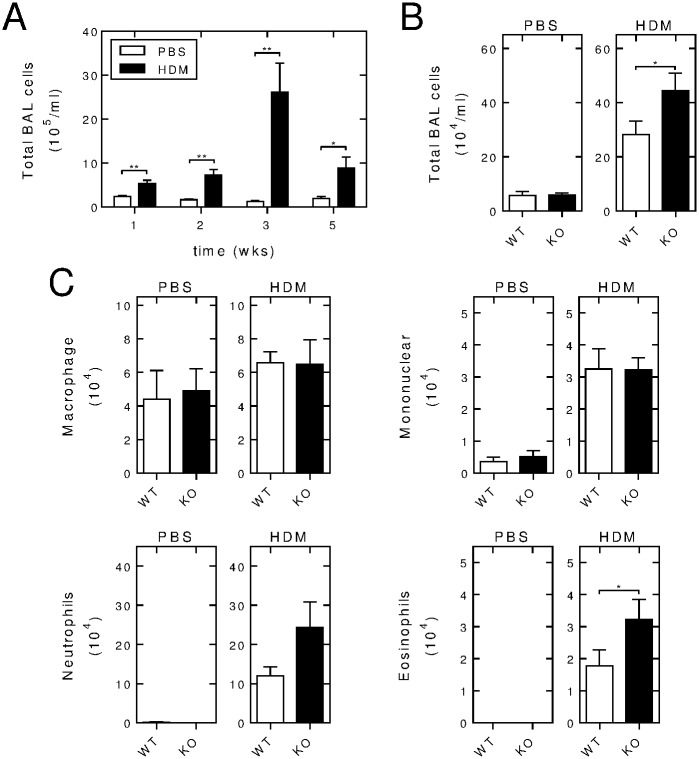
Effect of RGS2 deficiency on inflammatory cells in the BAL fluid. A, Wild type C57/Bl6 mice were subjected to *i*.*n*. instillation of PBS or HDM (25 μg) 3 times/week for 1, 2, 3 or 5 weeks. BAL fluid was collected and total cell counting was performed. Data (*N* = 4–7 for PBS- and 6–11 for HDM-exposed) are plotted as means ± SE. B, Wild type (WT) and *Rgs2*^-/-^ (KO) mice were subjected to *i*.*n*. treatment with PBS (left panel) or HDM (25 μg) (right panel) 3 times/week for 3 weeks. BAL fluid was collected for total cell counting. Data (*N* = 14 for PBS-exposed wild type and *Rgs2*^-/-^, *N* = 23 for HDM-exposed wild type and 18 *Rgs2*^-/-^) are plotted as mean ± SE. C, Wild type and *Rgs2*^-/-^ mice were exposed as in B. Cells in the BAL fluid were pelleted onto glass slides by cyto-centrifugation, stained with Diff-Quik, prior to differential cell counting of at least 400 cells per slide. Percentages of macrophages, mononuclear cells, neutrophils and eosinophils were converted into absolute numbers using the respective total cell counts. Data (*N* = 8 PBS-exposed wild type and *Rgs2*^-/-^, *N* = 17 HDM-exposed wild type and 12 *Rgs2*^-/-^) are plotted as means ± SE. Significance was tested between wild type and *Rgs2*^-/-^ groups by unpaired Mann Whitney U test. * *P* < 0.05, ** *P* < 0.01.

In the HDM-expose mice, cell counts in the BAL fluid revealed significantly increased total cell numbers at 1, 2, 3 and 5 weeks compared to time-matched PBS-treated control animals (Fig 5A). This effect appeared to be maximal 3 weeks post-HDM exposure and this time was used to compare wild type and *Rgs2*^-/-^ animals following the PBS or HDM instillation protocol. Following 3 weeks of *i*.*n*. PBS exposure there was no difference in the BAL fluid total cell counts between the wild type and *Rgs2*^-/-^ animals (Fig 5B, left panel). Following intranasal HDM instillation, total cell numbers were significantly increased in the BAL fluid of *Rgs2*^-/-^ animals compared to wild type animals (Fig 5B, right panel). Differential cell counting showed that the cells in the BAL fluid from PBS-exposed animals were predominantly macrophage and that this was unchanged between the wild type and *Rgs2*^-/-^ animals (Fig 5C). Following *i*.*n*. HDM, there were markedly increased numbers of mononuclear cells, as well as both neutrophils and eosinophils, with a modest increase in macrophage numbers. Comparison between the wild type and knock-out animals showed no effect of RGS2 loss on HDM-induced recruitment of macrophage or mononuclear cells to the BAL fluid. However, in the HDM-exposed animals there were elevated numbers of neutrophils and significantly increased eosinophil numbers in the BAL fluid from *Rgs2*^-/-^ animals compared to wild-type animals (Fig 5C). Thus, the overall increase in the total inflammatory cell infiltration observed in the BAL fluid from HDM-exposed *Rgs2*^-/-^ animals (Fig 5B, right panel) was primarily due to granulocytes.

### Effect of RGS2 deficiency on the expression of inflammatory genes

**Fig 6:**
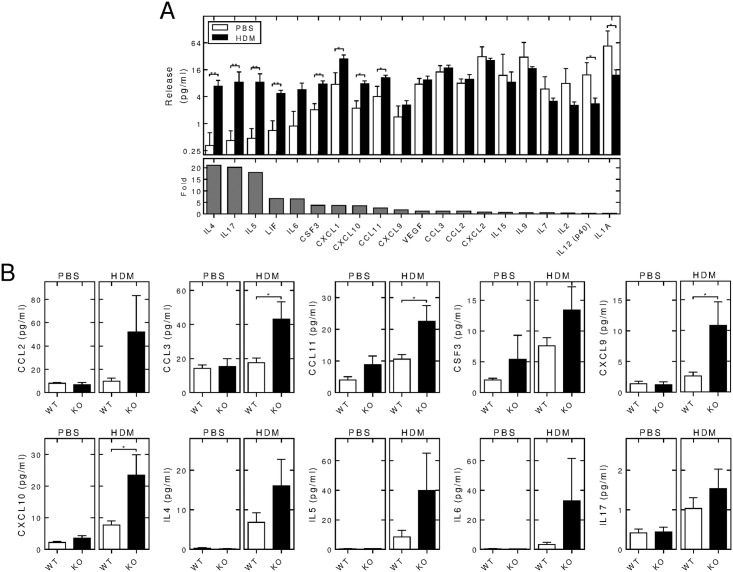
Effect of RGS2 deficiency on cytokines and chemokines in BAL fluid of PBS- and HDM-exposed animals. A, Wild type C57/Bl6 mice were subjected to i.n. instillation of PBS or HDM (25 μg) 3 times/week for 3 weeks. BAL fluid supernatants were collected and cytokine/chemokine levels quantified using a mouse cytokine/chemokine multiplex Luminex array. Protein release (pg/ml) is shown (upper panel) ranked according to the relative fold induction by HDM (lower panel). Data (*N* = 5 for PBS- and 8 for HDM-treated groups) are plotted as means ± SE. Significance was tested between PBS and HDM exposures by Mann Whitney U test. B, Wild type (WT) and *Rgs2*^-/-^ (KO) mice were subjected to *i*.*n*. exposure to PBS (left panels) or HDM (25 μg) (right panels) 3 times/week prior to Luminex analysis as in A. Data (*N* = 5 for PBS-treated wild type and *Rgs2*^-/-^, *N* = 8 for HDM-treated wild type and 9 for *Rgs2*^-/-^) are plotted as means ± SE. Significance was tested between wild type and *Rgs2*^-/-^ groups by Mann Whitney U test. * *P* < 0.05, ** *P* < 0.01.

Release of cytokines, chemokines and growth factors was examined in the BAL fluid from wild type animals exposed to PBS or HDM for 3 weeks. Expression of CCL4, CCL5, CSF1, CSF2, IFNγ, IL1β, IL3, IL10, IL12 (p70), IL13 and TNF was below the limit of detection of the Luminex assay in greater than 40% of the HDM-treated animals (wild-type and *Rgs2*^-/-^). These mediators were therefore considered absent from the BAL fluid. The remaining factors were produced at low picogram (2–64 pg/ml) levels and are shown ranked according to their relative increase in expression between the HDM- and PBS-exposed groups (Fig 6A). Levels of IL4, IL17, IL5, LIF, IL6, CSF3, CXCL1, CXCL10 and CCL11 were increased in the HDM-exposed animals compared to PBS-controls. Conversely, expression of IL12 (p40) and IL1A were reduced in HDM-exposed animals compared to PBS control animals.

Comparison between wild-type and *Rgs2*^-/-^ animals following PBS exposure revealed no differences in expression for the cytokines/chemokines and growth factors measured (Fig 6B). However, in the HDM animals there was evidence for increased release of numerous cytokines/chemokines in the *Rgs2*^*-/-*^ animals compared to wild-type (Fig 6B). This effect was significant for CCL3, CCL11, CXCL9 and CXCL10, with CCL2, CSF3, IL4, IL5, IL6 and IL17 all showing trends towards increased expression in the *Rgs2*^-/-^ animals. Conversely, while CXCL1 and LIF both showed increased release following HDM exposure, there were no significant differences between the wild type and *Rgs2*^-/-^ animals (data not shown). Equally, it should be noted that a number of the mediators, for example CCL2 and CCL3, were not apparently affected by HDM exposure, yet showed enhanced expression in the HDM-exposed *Rgs2*^*-/-*^ animals relative to HDM-exposed wild type animals (Fig 6B). Such data are suggestive of an enhanced inflammatory axis in the *Rgs2*^-/-^ mice.

**Fig 7:**
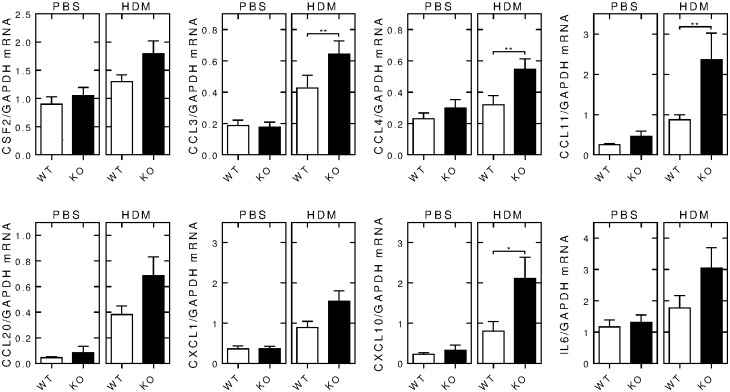
Effect of RGS2 deficiency on inflammatory mRNA expression in PBS- and HDM-exposed animals. Wild type (WT) and *Rgs2*^-/-^ (KO) mice were subjected to intranasal exposure to PBS (left panels) or HDM (25 μg) (right panels) 3 times/week for 3 weeks. RNA was extracted from the right lungs and mRNA analyzed by qPCR. Data (*N* = 23 for PBS-exposed wild type and 22 for *Rgs2*^-/-^, N = 34 for HDM-exposed wild type and 29 for *Rgs2*^-/-^), normalized to GAPDH mRNA, are plotted as means ± SE. Significance was tested between wild type and *Rgs2*^-/-^ groups by Mann Whitney U test. * *P* < 0.05 and ** *P* < 0.01.

Expression of various inflammatory gene mRNAs was examined by qPCR in RNA from the lungs of wild-type and *Rgs2*^-/-^ animals. While there were no differences in mRNA expression between the wild type and *Rgs2*^-/-^ animals following PBS instillation, in the HDM-exposed groups, of the eight mRNAs tested all showed elevated expression in the *Rgs2*^-/-^ animals compared to wild-type animals. This effect reached significance for CCL3, CCL4, CCL11 and CXCL10 (Fig 7).

### Effect of HDM instillation on RGS2 expression in the mouse lung

**Fig 8:**
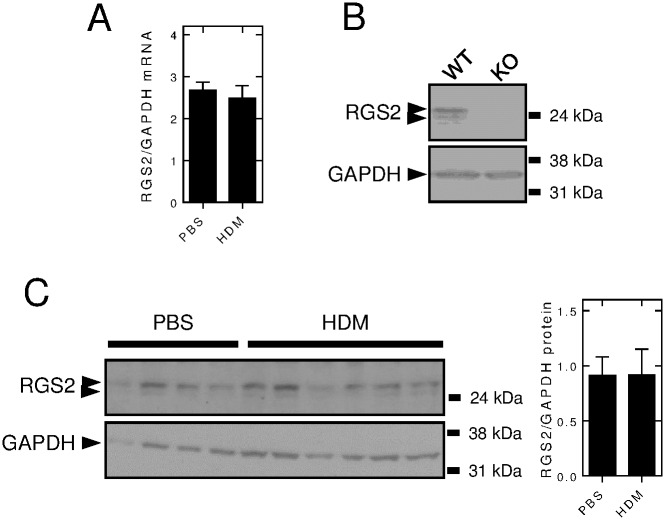
Effect of HDM instillation on *Rgs2* mRNA and RGS2 protein expression. A, Wild type mice were subjected to *i*.*n*. exposure with PBS or HDM (25 μg) 3 times/week for 3 weeks. RNA was extracted from the right lungs and RGS2 and GAPDH mRNA expression analyzed by qPCR. Data (*N* = 17 for PBS-exposed, *N* = 29 for HDM-exposed), normalized to GAPDH, are plotted as means ± SE. B, Total lung protein, prepared from wild type (WT) and *Rgs2*^-/-^ (KO) animals, was subjected to western blot analysis for RGS2 and GAPDH. Blots representative of at least 10 independent animals for each genotype are shown. C, Wild type mice were subjected to *i*.*n*. treatment with PBS or HDM (25 μg) 3 times/week and total lung proteins were subjected to western blot analysis for RGS2 and GAPDH. Data for 4 PBS and 6 HDM-exposed animals are shown (left panel). Following densitometric analysis of these data, RGS2 expression was normalized to GAPDH and is plotted as mean ± SE (right panel). Protein size markers (kDa) are shown to the right of each blot.

Wild type animals instilled with *i*.*n*. PBS or HDM for 3 weeks were analysized for RGS2 expression. Relative to GAPDH mRNA, there was no effect of the HDM exposure protocol on RGS2 mRNA expression (Fig 8A). Western blotting for RGS2 identified an immunoreactive doublet band that migrated at around 24 kDa and which was not present in *Rgs2*^-/-^ animals (Fig 8B). Accordingly, both bands were designated as RGS2 and in subsequent analysis, the expression of this doublet was not modified by HDM exposure, when compared to PBS control (Fig 8C). These data show no effect of the HDM exposure protocol on the expression of RGS2 mRNA or protein in the mouse lung.

## Discussion

The consequences of RGS2 deficiency in a murine model of airways inflammation induced by 3 weeks of *i*.*n*. HDM exposure are described and the data are summarized as S2 Table. As previously reported [27], HDM inhalation reduced lung function, evidenced by increased airways resistance and reduced compliance, and elicited a mixed granulocytic inflammation with attendant mucus hypersecretion. In terms of lung function, compared to wild-type, the *Rgs2*^-/-^ animals demonstrated AHR in both the PBS- and the HDM-exposure groups following challenge with methacholine, an agonist that acts at Gαq-coupled muscarinic M_3_ receptors to mediate ASM contraction [36, 37]. This involved not only absolute reductions in lung function, but also an enhanced, or exaggerated, propensity to lose lung function in the *Rgs2*^-/-^ animals. Thus, RGS2 is protective against a contractile agonist both in control and inflamed lungs. Similarly, baseline resistance was modestly elevated in the HDM-exposed group compared to PBS control. Given that RGS2 expression is present, indeed unaltered in the HDM-exposed animals, these data demonstrate a tonic protective role for RGS2 in HDM-induced inflammation. Furthermore, lack of change in RGS2 expression following HDM exposure means that other mechanisms must explain the observed HDM-induced deterioration in lung function. Since inflammation induced by HDM exposure will stimulate pro-inflammatory and contractile mediator release, as well as receptor expression [2], it is likely that such events are responsible for the increase in baseline resistance in the absence of the protective effects of RGS2. Similarly, RGS2 deficiency increased airways stiffness, i.e. reduced compliance, and this occurred in both the PBS control and the HDM-treated animals, both at baseline and following methacholine challenge. Such data are consistent with the known effects of RGS2 loss in the vasculature [24, 38]. Furthermore, these data add to previous reports that RGS2 is bronchoprotective in normal healthy lungs and in an ovalbumin model of lung inflammation [13, 14]. Equally, the recent finding that RGS2 mRNA expression is induced in the human airways 6 h following budesonide inhalation supports the concept that enhanced RGS2 expression, due to ICS alone, or possibly due to the synergy produced by an ICS/LABA combination therapy, may contribute towards the improved lung function produced by these therapeutic interventions [9, 13, 17].

Examination of various inflammatory indices revealed a peak in total BAL fluid cell counts and lung inflammation that occurred following 3 weeks of HDM exposure. Comparing the wild-type and *Rgs2*^-/-^ animals revealed a significantly enhanced cellular infiltration into the BAL fluid that was primarily accounted for by granulocytes. While inflammation scores obtained from analysis of H&E stained sections showed no obvious effect of RGS2 deficiency, enhanced expression of some cytokines and chemokines provides support for a modest anti-inflammatory role of RGS2. Reasons for this discrepancy are currently unclear, but may involve differences in the timing of these different aspects of the inflammatory response. Consistent with the increase in, at least some markers of inflammation, there was a trend towards increased ASM thickness in the HDM-exposed *Rgs2*^-/-^ animals compared to HDM-exposed wild type mice. Furthermore, the ASM thickness in the HDM-exposed *Rgs2-/-* mice was significantly greater than that in PBS-exposed wild type animals. Given, that most studies of airways remodelling utilise longer treatment protocols [25], it is possible that, with continued HDM exposure, a more prominent remodelling may have occurred. However, the current study was conducted to determine the role of RGS2 in HDM-induced inflammation and any effects on airways remodelling will need to be addressed separately. Nevertheless, as ASM thickness, or mass, was increased in the HDM-exposed *Rgs2*^-/-^ animals when compared to wild type, PBS-exposed animals this indicative of a protective role for RGS2 against inflammation-induced remodelling. This conclusion is consistent with data obtained in an ovalbumin model of airways inflammation [14]. Similarly, in pilot studies, HDM exposure produced a peak in PAS staining that was maximal at 3 weeks. Manual scoring of PAS staining, as well as morphometric analysis of the percentage of the airways epithelium that stained PAS positive, failed to reveal differences in the HDM-induced mucus hypersecretion between the wild type and *Rgs2*^-/-^ animals. These data were also supported by analysis of mucin gene expression, MUC5AC and MUC5B, which were both increased by HDM in a manner that was unaffected by RGS2 deficiency. While the expression of IL13 is strongly implicated in mucus hypersecretion, and goblet cell hyperplasia [39], in the current model, IL13 expression was below the limits of detection in the BAL fluid for the majority (wild type: 7 of 9; *Rgs2*^-/-^: 6 of 9 animals) of the HDM-exposed animals that were tested. Other mechanisms are therefore likely to be responsible for driving mucus hypersecretion. Nevertheless, expression of various other inflammatory cytokines, including IL4, IL5 and IL17 were increased with HDM exposure. Likewise, core inflammatory gp130 receptor cytokines (LIF, IL6) showed increased expression along with chemokines (CXCL10, CXCL11), including the potent neutrophil chemoattractant, CXCL1, and growth factors (CSF3) for granulocytes and lymphocytes. The presence of these mediators is collectively consistent with the mixed neutrophilic/eosinophilic inflammatory infiltrate observed in the BAL fluid of the HDM exposed mice. Furthermore, expression of IL12 (p40) was downregulated and this may be considered as evidence of reduced Th1 type responsiveness. In terms of the effects of RGS2 loss, these were relatively modest. While the HDM-induced release of CXCL1 was not modified in the *Rgs2*^-/-^ mice, direct comparison of *Rgs2*^-/-^ and wild-type animals revealed a trend towards enhanced HDM-induced expression of multiple cytokines and chemokines at both the protein and mRNA levels. Most prominent, and significantly, were the release of CCL3, CCL11 along with CXCL9 and CXCL10 into the BAL fluid. These chemokines act on the receptors CCR1, CCR3, CCR5 and CXCR3 that are present on lymphocytes (T, B and NK cells), granulocytes (neutrophils and eosinophils), macrophage, dendritic cells and mast cells [40, 41]. Thus, while the overall inflammatory response to HDM exposure was relatively unchanged, there is clear evidence for a protective, anti-inflammatory effect of RGS2. This may, in part, explain the enhanced granulocyte (neutrophil/eosinophil) numbers observed in the BAL fluid of the HDM-exposed *Rgs2*^-/-^ animals. Furthermore, it is tempting to speculate that, in more severe neutrophilic asthma, the above effects of RGS2 could play a role in the enhanced clinical effectiveness of ICS/LABA combinations, compared to ICS as a monotherapy [42].

In exploring the way(s) by which RGS2 may modulate inflammatory responses, it is necessary to consider the pathways and processes by which HDM may induce inflammation. Possibly the most obvious, is that proteases in the HDM, for example the major allergen, Der p 1, leads to the activation of PARs [2]. In this regard, PAR2 is particularly implicated in the development of allergic inflammation in response to allergens, such as ovalbumin, cockroach and HDM [30–32, 43]. Indeed, PAR2 is highly expressed in the bronchial epithelium and agonists and activators of PAR2, including Der p 1, produce a variety of inflammatory responses, including the release of cytokines, other inflammatory mediators and matrix-metalloproteases [29, 44–48]. Furthermore, expression of PAR2 is increased on monocytes from asthmatic patients [49]. However, while such data support a role for PAR2 in promoting inflammatory responses and inflammation [34], a more modulatory role for PAR2 may be possible. For example, while PAR2 activators induce the release of proteases from mast cells, this results in the proteolytic degradation of inflammatory cytokines [50]. Equally, agonism of Gαq-coupled receptors is associated with phospholipase activation, the release of arachidonic acid and widespread generation of prostaglandins (PGs), including PGE_2_ from ASM and epithelial cells [51, 52]. While the production of PGD_2_, by reducing NK cell populations, may promote eosinophilic inflammation [53], PGE_2_, which is released from epithelial cells via a PAR2-dependent [Ca^2+^]_i_ release-activated Ca^2+^ channel mechanism [54], relaxes ASM and may thus provide bronchoprotection [55, 56]. However, while the anti-inflammatory effect of RGS2 is modest, there was clearly impaired lung function in the *Rgs2*^-/-^ animals suggesting that possible increases in PGE_2_-dependent bronchoprotection were not overriding.

Other pathways that promote PAR2-dependent inflammation and which would not be subject to control via RGS2 include signalling, and the induction of inflammatory cytokines, via β-arrestin-dependent pathways [57]. Indeed, HDM Der p 1 has been shown to induce IL8 expression via PAR2-independent mechanisms [58]. For example, proteases present in HDM may lead to epithelial cell activation and pro-inflammatory effects by causing damage including; loss of tight junctions and/or reduced membrane integrity and barrier function [2]. Equally, in addition to various proteases, HDM contains a wide variety of additional stimuli, including lipopolysaccharide and D-glucans, which will activate various pattern recognition receptors [2, 59]. Thus, there is a considerable role for non-PAR components of the immune system, in particular innate immune responses, in the overall effect produced by HDM [33]. Indeed, TLR/IL1 receptors are major effector pathways in HDM-induced allergic inflammation and TLR4 is specifically implicated [60–63]. As such, HDM-induced inflammatory responses and the resultant inflammation may occur via a number of PAR-independent, and therefore Gαq-independent processes, for which protective roles by RGS2 are not therefore predicted. Nevertheless, the secondary release of contractile and pro-inflammatory mediators, for example certain prostaglandins and leukotrienes may, by acting on Gαq-coupled receptors produce responses that should be prevented, or at least reduced, by RGS2.

In summary (See S2 Table), our data confirm in a model of HDM-induced airways inflammation that RGS2 is bronchoprotective. Similarly, the current data support a modest protective effect of RGS2 in respect of HDM-induced inflammation and potentially remodelling, and this is consistent with a partial role of Gαq-coupled GPCRs, such as PAR2, in generating inflammatory responses in the HDM model. Importantly, the knowledge that RGS2 expression is induced in the human airways following ICS inhalation and the fact that in primary airway epithelial and smooth muscle cells glucocorticoids and LABAs synergistically induce RGS2 expression is also consistent with a therapeutically beneficial role of RGS2 expression in asthma [9, 13, 17]. Such effects are likely to operate alongside the known effects of other glucocorticoid-induced gene products [64]. For example, DUSP1, which acts to reduce MAPK signalling, is induced by glucocorticoids and LABAs and may also reduce expression of inflammatory genes and provide bronchoprotection [65–70]. In seeking to design improved glucocorticoids, and/or ICS/LABA combination therapies, we propose that strategies seeking to maximize expression of RGS2, and other beneficial gene products, may be therapeutically advantageous [42, 71].

## Supporting Information

S1 Table Primers and probes used for genotyping and qPCR. (DOCX)

S2 Table Overview data summary. (DOCX)
