# Supplementary material for: Protective Roles for RGS2 in a Mouse Model of House Dust Mite-Induced Airway Inflammation
Source: PLoS One. 2017 Jan 20;12(1):e0170269. doi: 10.1371/journal.pone.0170269 (PMC5249169; doi:10.1371/journal.pone.0170269)
Supplement: S1 Table — (DOCX) [file pone.0170269.s001.docx]

**Supporting Information 1 - Table**

**A, Primers and Probes used for genotyping**

The sequences (5’-3’) are shown for the WT and KO genotyping primers and Taqman probes. The probes were synthesized by Applied Biosystems and the primers were synthesised by the DNA synthesis laboratory at the University of Calgary.

| Type | Name | Sequences (with probe and quencher as appropriate) |
| --- | --- | --- |
| WT gene probe | WTP2mRGS2 | VIC-CTGTAGTCCTAAGAACG-MGB |
| KO gene probe | KOP2mRGS2 | 6FAM-ATCCCCCAATTCTACCG-MGB |
| WT Forward primer | WTF4 mRGS2 | GTTTACTGTGTGCAAGGGTGT TG |
| WT Reverse primer | WTR3 mRGS2 | CAGTTTCAGTGATACGTGACACACTAG |
| KO Forward primer | KOF4 mRGS2 | CAGACTGCCTTGGGAAAAGA |
| KO Reverse primer | KOR3 mRGS2 | CAGTTTCAGTGATACGTGACACAC TAG |

**B, Primers used for qPCR analysis**

Forward (F) and reverse (R) primer sequences (5’-3’) are shown in addition to the accession number for each gene. For genes with more than one splice variant, primers were designed to pick up all variants. All primers were designed using Primer Express software (Applied Biosystems) and were synthesised by the DNA synthesis laboratory at the University of Calgary.

| Target gene | Accession Number | Primer Sequences |
| --- | --- | --- |
| *Ccl3* | NM_011337.2 | F: TCTTCTCAGCGCCATATGGA  R: TCCGGCTGTAGGAGAAGCA |
| *Ccl4* | NM_013652.2 | F: CAGCACCAATGGGCTCTGA  R: TGCCGGGAGGTGTAAGAGAA |
| *Ccl5* | NM_013653.3 | F: TGCCCACGTCAAGGAGTATTT  R: ACTTCTTCTCTGGGTTGGCAC |
| *Ccl11* | NM_011330.3 | \| F: ATCCCAACTTCCTGCTGCTTT \| \| --- \| \| R: AGATCTCTTTGCCCAACC TGG \| |
| *Ccl20* | NM_016960.2  NM_001159738.1 | F: GTGGGTTTCACAAGACAGATG  R: TTTTCACCCAGTTCTGCTTTG |
| *Cxcl1* | NM_008176.3 | \| F: CAATGAGCTGCGCTGTCAGT \| \| --- \| \| R: CTGGATGTTCTTGAGGTGAATCC \| |
| *Cxcl2* | NM_009140.2 | F: TCAAGAACATCCAGAGCTTGAG  R: TTCAGGGTCAAGGCAAACTT |
| *Cxcl10* | NM_021274.2 | F: CCAAGTGCTGCCGTCATTTT  R: TTCAAGCTTCCCTATGGCCC |
| *Cxcl15* | NM_011339.2 | \| F: TCGAGACCA TTTACTGCAACAGA \| \| --- \| \| R: TTGGGCCAACAGTAGCCTTC \| |
| *Gapdh* | NM_001289726.1  NM_008084.3 | \| F: AGCCCATCACCATCTTCCAG \| \| --- \| \| R: GATGACCCTTTTGGCTCCAC \| |
| *Csf2* | NM_009969.4 | \| F: TCAAAGAAGCCCTGAACCTCC \| \| --- \| \| R: GTGAAATTGCCCCGTAGACC \| |
| *Ifng* | NM_008337.4 | \| F: CCACGGCACAGTCATTGAAA \| \| --- \| \| R: CTGCAGGATTTTCATGTCACCA \| |
| *Il6* | NM_031168.2  NM_001314054.1 | F: TGTTCTCTGGGAAATCGTGGA  R: TGCAAGTGCATCATCGTTGTTC |
| *Muc5ac* | NM_010844.1 | F: CCATCATCCCACCTCTGAAGAC  R: CCTGGTATGTCCTGGGTTGAA |
| *Muc5b* | NM_028801.2 | \| F: CTCACCGGAGACAGTCAGAGA \| \| --- \| \| R: GTGCTGCACACTCTCCCATT \| |
| *Rgs2* | NM_009061.4 | \| F: GAGGAGAAGCGGGAGAAAATG \| \| --- \| \| R: GCTCAAACGGGTCTTCCAATC \| |
| *Tgfb1* | NM_011577.2 | F: GCCCGAAGCGGACTACTATG  R: ACTGCTTCCCGAATGTCTGA |
| *Tnf* | NM_013693.3  NM_001278601.1 | \| F: ACTGAACTTCGGGGTGATCG \| \| --- \| \| R: ACAGGCTTGTCACTCGAATTTTG \| |
