# Supplementary material for: Protective Roles for RGS2 in a Mouse Model of House Dust Mite-Induced Airway Inflammation
Source: PLoS One. 2017 Jan 20;12(1):e0170269. doi: 10.1371/journal.pone.0170269 (PMC5249169; doi:10.1371/journal.pone.0170269)
Supplement: S2 Table — (DOCX) [file pone.0170269.s002.docx]

**Supporting Information 2 - Table**

**Overview data summary**

Data are summarized from the main manuscript. Abbreviations and other terms are as used in the main manuscript. Wild type = WT; *Rgs2*^-/-^ knockout = KO.

| **Experimental**  **parameters**  **measured** | **Experimental comparisons** | | |
| --- | --- | --- | --- |
|  | **Baseline effect of *Rgs2*^-/-^**  Genotype: WT vs KO  Exposure: All PBS | **Effect of HDM exposure**  Genotype: All WT  Exposure: PBS vs HDM | **HDM exposed & effect of *Rgs2*^-/-^**  Genotype: WT vs KO  Exposure All HDM |
| **Lung function**  Resistance  Compliance | ↑ in KO  ↓ in KO | ↑ with HDM  ↓ with HDM | ↑ in KO  ↓ in KO |
| **Histology**  Inflammatory Score (H&E)  ASM thickness  PAS and mucin expression | No effect of KO  No effect of KO  No effect of KO | ↑ with HDM  trend ↑ with HDM  ↑ with HDM | No effect of KO  trend ↑ with KO  No effect of KO |
| **Cells and cytokines**  Total BAL fluid cell counts  BAL fluid cyto-/chemokines | No effect of KO  No effect of KO | ↑ with HDM  ↑ with HDM | ↑ granulocytes in KO  ↑ for some in KO |
